# Alterations in glycolytic/cholesterogenic gene expression in hepatocellular carcinoma

**DOI:** 10.18632/aging.103254

**Published:** 2020-06-01

**Authors:** Jianwen Jiang, Qiuxian Zheng, Weiwei Zhu, Xinhua Chen, Haifeng Lu, Deying Chen, Hua Zhang, Min Shao, Lin Zhou, Shusen Zheng

**Affiliations:** 1 Department of Health Management, The First Affiliated Hospital, Zhejiang University School of Medicine, Hangzhou 310003, China; 2 Department of Hepatobiliary and Pancreatic Surgery, The First Affiliated Hospital, Zhejiang University School of Medicine, Hangzhou 310003, China; 3 Key Laboratory of Combined Multi-Organ Transplantation, Ministry of Public Health, The First Afﬁliated Hospital, Zhejiang University School of Medicine, Hangzhou 310003, China; 4 Collaborative Innovation Center for Diagnosis and Treatment of Infectious Diseases, The First Affiliated Hospital, Zhejiang University School of Medicine, Hangzhou 310003, China; 5 State Key Laboratory for Diagnosis and Treatment of Infectious Diseases, The First Affiliated Hospital, Zhejiang University School of Medicine, Hangzhou 310003, China

**Keywords:** hepatocellular carcinoma, metabolic classification, glycolysis, cholesterogenic, molecular mechanism

## Abstract

Metabolic reprogramming is a hallmark of tumors, including hepatocellular carcinoma (HCC). We used data from The Cancer Genome Atlas and the International Cancer Genome Consortium to assess the alterations in glycolytic and cholesterogenic genes in HCC and to determine their association with clinical features in HCC patients. Based on the gene expression profiles from these databases, we established four subtypes of HCC: cholesterogenic, glycolytic, mixed, and quiescent. The prognosis of the cholesterogenic subgroup was poorer than that of the glycolytic group. Tumors in the glycolytic group were more sensitive to chemotherapy. We also explored the relationships between these metabolic subtypes and previously established HCC subgroups. Glycolytic gene expression correlated strongly with poorer prognostic gene expression in the Hoshida classification of HCC. Whole-genome analyses indicated that aberrant amplification of *TP53* and *MYC* in HCC were associated with abnormal anabolic cholesterol metabolism. The mRNA levels of mitochondrial pyruvate carriers 1 and 2 differed among the HCC metabolic subtypes. In a bioinformatics analysis we identified genomic characteristics of tumor metabolism that varied among different cancer types. These findings demonstrate that metabolic subtypes may be valuable prognostic indicators in HCC patients.

## INTRODUCTION

Hepatocellular carcinoma (HCC) is the most common type of primary liver cancer, accounting for 70-90% of cases [1]. The occurrence of HCC is strongly associated with hepatitis viral infections, alcohol abuse and aflatoxin contamination [2]. Because HCC is difficult to diagnose early, and is also highly malignant and insensitive to chemoradiotherapy, it is a serious threat to human health. The latest epidemiological surveys have demonstrated that HCC is the second leading cause of cancer death among men worldwide and the sixth leading cause of cancer death among men in developed countries [3, 4]. Although advances in HCC treatment have been made in recent years, HCC recurrence and metastasis are still key determinants of the long-term prognosis of patients, and are the main obstacles to patient survival [5].

The study of metabolic reprogramming in tumors has developed in recent years, and may provide a new method of eliminating tumor cells effectively [6, 7]. To satisfy the additional energy requirements for their proliferation and growth, tumor cells must reshape their metabolic pathways [8]. Tumor cells differ from normal cells in their metabolism of glucose, amino acids, fatty acids and nucleotides, which provide large amounts of energy and intermediates [9]. The metabolic reprogramming of tumor cells primarily involves hyperactive glycolysis and fatty acid synthesis. Key metabolic enzymes are upregulated in a variety of cancer types, including lung cancer [10], prostate cancer [11], kidney cancer [12] and lymphoma [13]. Using an online prediction tool, we previously illustrated that beta-lactamase expression correlated strongly with the expression of genes involved in lipid metabolism in HCC patients [14]. Although disruptions in certain signaling pathways are known to contribute to metabolic reprogramming in cancer, alterations in glycolipid metabolism have rarely been reported in liver cancer.

Otto Warburg first reported that liver cancer cells exhibited significantly greater glycolytic activity than normal hepatocytes, and proposed that rapidly proliferating tumor cells were powered by aerobic glycolysis [15]. This phenomenon has been observed in different tumor types, and has become fundamental to our understanding of tumor metabolism. Similarly, cholesterol levels are significantly higher in liver cancer than in healthy liver tissues [16]. Cholesterol is important for the formation of cell membranes and for the synthesis of bile acids, vitamin D and steroid hormones [17]. Previous studies have revealed that metabolism-related genes (including isoenzymes within specific pathways) exhibit an increased mutation rate in cancer patients and display heterogeneity among different cancer types [18, 19]. However, no systematic reports have been published to date on the relationship of abnormal glucose and lipid metabolism to the molecular mechanism, prognosis and treatment of HCC.

The lack of molecular subtyping for HCC tumors makes it impossible to screen patients for their suitability for targeted therapies. The latest guidelines for the diagnosis of primary liver cancer mainly describe its pathology in terms of gross and histological morphology, while only using immunohistochemical indexes to distinguish the source of the tumor cells (HCC, biliary cell carcinoma or mixed cell types). The molecular classification of liver cancer lags behind those of lung, breast and gastric cancers, and does not satisfy the requirements for clinically accurate treatment. Understanding the reprogramming of energy metabolism in liver cancer could provide a new strategy for subtyping HCC patients so that precise and targeted treatments can be developed to improve survival.

In the present study, we classified HCC patients into different subtypes based on their expression of genes involved in glycolysis and cholesterol synthesis. We explored the differences in survival and other clinical characteristics among the various metabolic subtypes of HCC, and identified carcinogenic molecular events in the different subtypes. We now propose a clinically feasible HCC-typing scheme, which may become a new tool to guide the targeted therapy of HCC.

## RESULTS

### Four metabolic subgroups of HCC were identified based on the dual analysis of glycolytic and cholesterogenic gene expression

**Figure 1:**
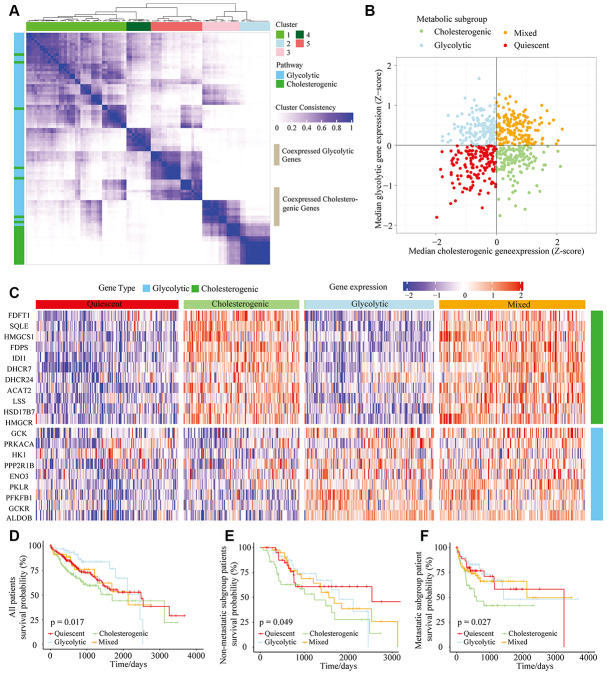
**The metabolic gene landscape of HCC based on glycolytic and cholesterogenic clusters.** (**A**) Heat map of consensus clustering (k=5) for glycolytic and cholesterogenic genes in resected and metastatic LIHC samples (n=610). (**B**) Scatter plot of the median levels of co-expressed glycolytic (x-axis) and cholesterogenic (y-axis) genes in each LIHC sample. Metabolic subgroups were assigned based on the relative levels of glycolytic and cholesterogenic genes. (**C**) Heat map of differential gene expression patterns in glycolytic and cholesterogenic gene clusters across subgroups. (**D**) Kaplan-Meier survival analyses of patients with all subtypes of LIHC; the log-rank test p value is shown. (**E**) Overall survival analyses in the metastatic subgroup of LIHC patients; the log-rank test p value is shown. (**F**) Overall survival analyses in the non-metastatic LIHC cohort; the log-rank test p value is shown.

In total, 610 HCC tumor samples were included in this study (The Cancer Genome Atlas [TCGA], n = 373, and International Cancer Genome Consortium [ICGC], n = 237). Samples with < 30% were excluded. Reactome gene set enrichment analysis was applied to obtain the “glycolysis” (n = 29) and “cholesterol biosynthesis” (n = 72) gene sets. Unsupervised consensus clustering analysis was implemented to classify the two subgroups of significantly expressed glycolytic (n = 9) and cholesterogenic (n = 11) genes for further metabolic subtyping (Figure 1A).

We calculated the median levels of glycolytic and cholesterol-producing genes in each sample. Based on the co-expression of the two gene sets, we separated the gene profiles into four metabolic subtypes of HCC: the glycolytic subgroup (high expression of glycolytic genes and low expression of cholesterol synthesis genes), cholesterogenic (high expression of cholesterol synthesis genes and low expression of glycolytic genes), mixed (high expression of both cholesterol synthesis genes and glycolytic genes) and quiescent (low expression of both cholesterol synthesis genes and glycolytic genes) subgroups (Figure 1B). The levels of genes involved in glycolysis and cholesterol synthesis are illustrated in Figure 1C, along with the proportion of patients in each metabolic subgroup. The quiescent phenotype group contained the largest number of patients (332/610, 54.4%), followed by the cholesterogenic (164/610, 26.9%), glycolytic (65/610, 11%) and mixed subtypes (49/610, 8%).

To determine the relationship between the HCC metabolic subgroup and patient prognosis, we performed a statistical cluster analysis on HCC metabolic oncogene characteristics in metastatic and non-metastatic patients. There was no significant difference in the distribution of the metabolic subgroups between the metastatic and non-metastatic groups. However, there were significant differences in overall survival based on cholesterol-generating and glycolytic gene expression. The clinical outcomes were significantly worse in the cholesterol-generating subgroup than in the glycolytic group (p = 0.017) (Figure 1D). In both the non-metastatic and metastatic groups of HCC patients (Figure 1E and 1F, p = 0.049 and p = 0.027, respectively), there was a significant difference in survival between the cholesterogenic and glycolytic subgroups. Surprisingly, survival benefits were observed in patients with increased expression of glycolytic genes. These findings indicated that there are metabolic phenotypes associated with glycolysis/cholesterogenesis and prognosis in HCC.

### Relationship between the tumor genome metabolic subtype and the HCC subtype

**Figure 2:**
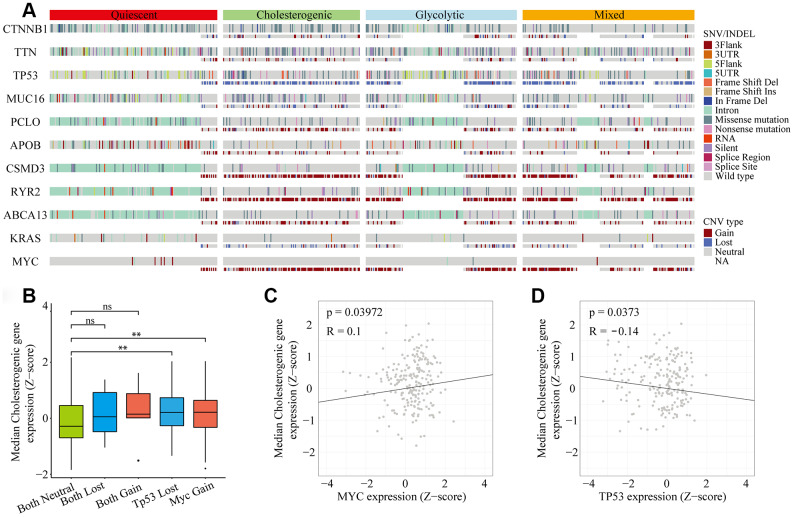
**Gene mutational landscape across metabolic subgroups of HCC.** (**A**) Oncoprint analysis indicating the distribution of SNVs, INDELs and CNVs of frequently mutated genes in LIHC across the metabolic subtypes. (**B**) Box plot of the median expression of cholesterogenic genes in samples with CNVs in *TP53* and/or *MYC*. (**C**) Scatter plot of the correlation between the median cholesterogenic gene expression and *MYC* expression. (**D**) Scatter plot of the relationship between the median cholesterogenic gene expression and *TP53* expression.

Metabolic reprogramming is now accepted as a hallmark of cancer [20, 21], and we found significant abnormalities in metabolic gene expression in HCC patients. We next investigated the frequency of single nucleotide variations (SNVs), insertion-deletion mutations (INDELs) and copy number variations (CNVs) [22] in genes associated with the different metabolic subtypes of liver hepatocellular carcinoma (LIHC) cohorts (Figure 2A). The mutational frequency of each gene did not differ significantly among the subtypes (Fisher’s exact test and Benjamini-Hochberg (BH) test-corrected, p > 0.05 after correction). However, the median levels of cholesterol synthesis genes were significantly greater in *MYC*-amplified and *TP53*-deleted samples than in samples without these alterations (Figure 2B). Cholesterogenic gene expression correlated positively with *MYC* mutations (p = 0.039, R = 0.1) (Figure 2C) and negatively with *TP53* mutations (p = 0.037, R = -0.14) (Figure 2D). These findings are consistent with the high mutational frequency of *TP53* and *MYC*, and support the notion that mutations in *TP53* and *MYC* promote tumor progression by inducing abnormal glucose utilization. Thus, tumors may be vulnerable to changes in glycolysis.

**Figure 3:**
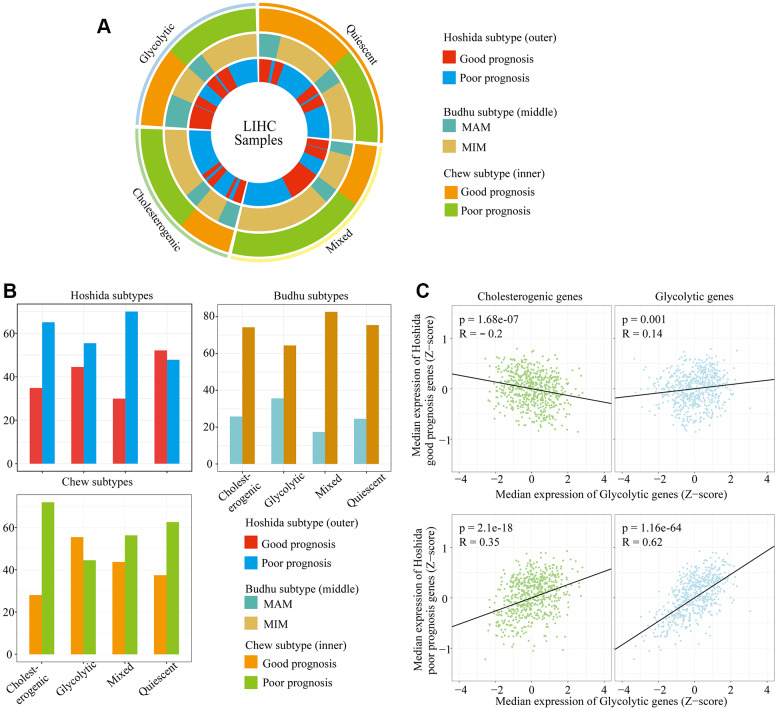
**The alignment of LIHC metabolic subgroups with known gene expression subtypes.** (**A**) Overlay of the metabolic gene profiles with LIHC expression subtypes based on the known classifications of Hoshida et al., Budhu et al. and Chew et al. (**B**) Bar plots of the proportion of LIHC expression subtypes in each metabolic subgroup. (**C**) Scatter plots depicting the correlations of glycolytic and cholesterogenic gene levels with prognostic gene levels in the Hoshida classification.

The relationship between survival-related features and gene expression in HCC has been illustrated in previous studies [23, 24]. Hoshida’s classification, Budhu’s metastasis-averse/inclined microenvironment (MAM/MIM) and Chew’s classification have been used to classify liver cancer according to the clinical prognosis (see Methods for details) (Supplementary Table 1). To explore the association between the glycolytic/cholesterogenic metabolic phenotypes and the tumor subtypes from the above-mentioned prognostic classification methods, we identified the tumor subtype and metabolic phenotype of each sample (Figure 3A). Most of the patients in the quiescent group had favorable prognoses according to the Hoshida classification (75.4%), while fewer patients in the mixed group (29.9%, adjusted p = 0.0025) and the cholesterogenic group (34.8%, adjusted p = 0.022) had favorable prognoses (Figure 3B). The number of MAM/MIM samples according to the Budhu classification differed significantly between the quiescent group and the mixed group. The number of samples with a poor prognosis according to the Chew classification differed significantly between the quiescent group and the glycolytic group.

We also analyzed the correlation of cholesterogenic/glycolytic gene expression with prognostic gene expression according to the Hoshida classification. The expression of genes associated with a poor prognosis correlated positively with the expression of genes in the cholesterol synthesis pathway. Likewise, the expression of genes associated with a good prognosis correlated negatively with the expression of genes in the cholesterol synthesis pathway. Glycolytic gene expression correlated positively with prognostic gene expression in both the better and poorer prognosis groups, but the correlation was stronger in the poorer prognosis group (Figure 3C). This was consistent with the significant association between cholesterogenic gene expression and a poorer survival prognosis. These data indicated that glycolysis and cholesterol synthesis are potential metabolic targets in patients with different HCC subtypes.

### Mitochondrial pyruvate carrier complex expression differed among the metabolic subtypes

The mitochondrial pyruvate carrier (MPC) complex in the inner membrane of the mitochondria transfers free pyruvate from the cytoplasm to the mitochondrial matrix [25]. Previous studies observed that MPC1 and MPC2 were different in metabolic pathways and promoted tumor glycolysis activity. This difference is important in lactic acid production [26, 27].

**Figure 4:**
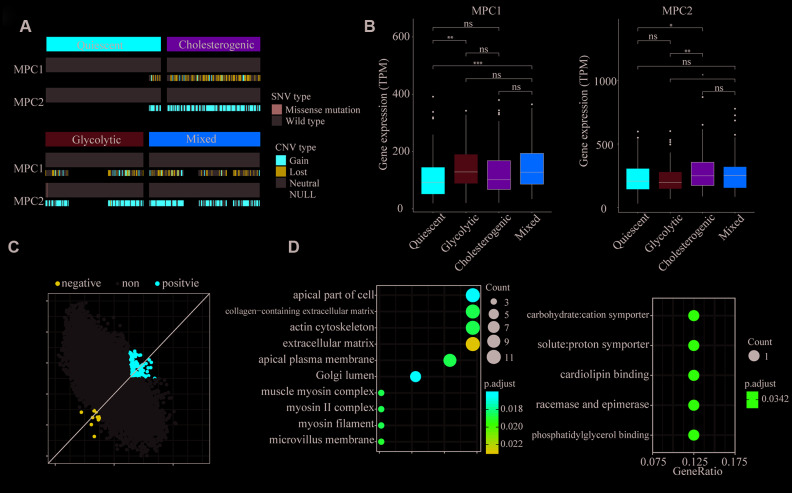
**Association of *MPC1* and *MPC2* expression with LIHC metabolic subgroups and cell signaling pathways.** (**A**) Oncoprint indicating the distribution of *MPC1* and *MPC2* SNVs and CNVs across the metabolic groups. Only one case was found with an SNV in *MPC2*. (**B**) Box plots of significant (p < 0.001) differences in *MPC1* and *MPC2* levels across the LIHC metabolic subgroups. (**C**) Scatter plot of the correlations of 25,483 genes with *MPC1* (x-axis) and *MPC2* (y-axis). In total, 168 genes correlated positively (Spearman correlation BH-adjusted p < 0.01) with both *MPC1* and *MPC2*, while 14 genes correlated negatively with both *MPC1* and *MPC2* (adjusted p < 0.01). (**D**) The most significantly enriched (hypergeometric test BH-adjusted p < 0.05) gene sets among the genes positively (left) and negatively (right) associated with *MPC1/2* expression.

To explore the association of *MPC1* and *MPC2* with the different metabolic phenotypes, we assessed the metabolic subpopulations for SNVs, INDELs, CNVs and mRNA expression changes in these genes. The CNVs in *MPC1* and *MPC2* differed significantly among the metabolic subgroups; however, the CNVs in *MPC1* were deletions in almost all of the HCC samples, while the CNVs in *MPC2* were mostly amplifications (Figure 4A). *MPC1* and *MPC2* mRNA levels differed significantly among the metabolic subgroups. *MPC2* expression was significantly lower in the glycolytic group than in the other groups, whereas *MPC1* expression was significantly greater in the glycolytic group. *MPC2* expression was significantly higher in the cholesterogenic group than in the quiescent group (Figure 4B). Thus, the dysregulation of mitochondrial pyruvate transport at the mRNA level may be associated with the metabolic tumor subtypes.

To find cellular pathways associated with *MPC1/2* expression, we performed a comprehensive correlation analysis between *MPC1/2* and all the other tested genes (n = 25,483). In total, 168 genes correlated positively with *MPC1/2*, while 14 genes correlated negatively with *MPC1/2* (Spearman correlation BH-corrected p < 0.01) (Figure 4C). The positively correlated genes were associated with extracellular matrix function (hypergeometric test, BH regulation p < 0.05). The pathways enriched in the negatively correlated genes were involved in carbon chain binding and phosphorylation (Figure 4D). These data suggested that MPC1 and 2 participate in cellular networks associated with tumor progression in HCC.

### Correlation between glycolytic and cholesterogenic gene expression in various cancers

**Figure 5:**
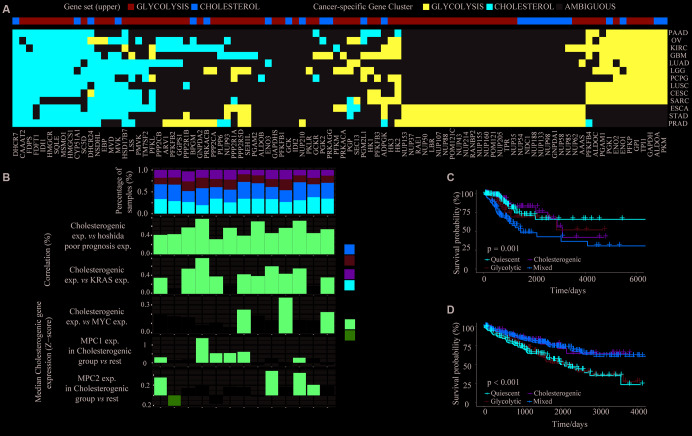
**The glycolytic and cholesterogenic gene profiles of other cancer types.** (**A**) Heat map depicting that glycolytic and cholesterogenic genes were robustly co-expressed when consensus clusters were applied to each individual cancer type. (**B**) Top: Bar plots showing the proportions of the metabolic subgroups across the various cancer types. Bottom: The correlation between cholesterogenic gene expression and the expression of Hoshida poor prognostic genes, *KRAS*, *MYC* and *MPC1/2* in each cancer type. Median glycolytic gene expression correlated positively (BH-adjusted p < 0.05) with basal-like gene expression in all cancer types. The correlation between *MPC1/2* expression and glycolytic gene expression was validated using Wilcoxon rank sum tests and BH correction. (**C**) Kaplan-Meier survival analysis curves depicting the differences in median overall survival across the metabolic subgroups in CESC. (**D**) Kaplan-Meier survival analysis curves demonstrating the differences in median overall survival in KIRC.

It is very important to identify cancers that have unique metabolic characteristics driven by their mutational environment and organ-specific enzyme expression [28]. To identify the dysregulated routes of metabolism in different cancers, we performed cluster analyses of glycolytic and cholesterogenic gene expression in 26 cancer types (tumor content ≥ 30%) from TCGA (Supplementary Table 2). Gene co-expression pathway-specific profiles and hub genes were identified via network topology analysis in 13 cancer types. Metabolism-related genes were co-expressed in most tumor types. However, due to the differential co-expression of glycolytic and cholesterogenic genes, some genes were only co-expressed in a few cancer types, indicating that certain genes contribute to the tumor metabolic program in a cell-type-specific manner (Figure 5A). Cholesterogenic gene expression correlated positively with poor prognostic gene expression in the Hoshida classification system, not only in HCC (Spearman correlation BH-corrected p < 0.05), but also in a range of other tumor types (Figure 5B). For some cancers, the median cholesterogenic gene expression correlated positively with *KRAS* expression (cervical squamous cell carcinoma [CESC], glioblastoma multiforme [GBM], kidney renal clear cell carcinoma [KIRC], brain lower grade glioma [LGG], lung squamous cell carcinoma [LUSC], ovarian serous cystadenocarcinoma [OV], pancreatic adenocarcinoma [PAAD], pheochromocytoma and paraganglioma [PCPG], prostate adenocarcinoma [PRAD] and stomach adenocarcinoma [STAD]) or *MYC* expression (PCPG, STAD and LUSC) (BH-corrected p < 0.05). The expression of *MPC1* was significantly upregulated in the cholesterogenic group in KIRC, LGG, lung adenocarcinoma (LUAD) and LUSC, and the expression of *MPC2* was significantly upregulated in the cholesterogenic group in PAAD, sarcoma (SARC) and CESC (Figure 5B), similar to our findings in HCC. Further research is needed to explore the mRNA levels of these genes.

The potential associations between specific metabolic genes and the clinical features of various cancers were analyzed. The survival rates differed significantly among patients in the four metabolic subtypes in CESC (p = 0.001) (Figure 5C) and KIRC (p < 0.001) (Figure 5D). In CESC, the overall survival rate was significantly lower in the mixed subgroup than in the cholesterogenic group. In KIRC, the prognosis was worse in the glycolytic and quiescent groups than in the mixed and cholesterogenic groups. Thus, our model has provided novel insights into the molecular features of multiple cancers and revealed the tumor specificity of metabolic subtype gene expression.

## DISCUSSION

HCC is a heterogeneous cancer that lacks effective treatment methods. Metabolic gene assessment may be a useful tool for investigating the metabolic abnormalities that characterize cancer cells [29]. During tumorigenesis, metabolic pathways are often reorganized to adapt to malignancy, and the corresponding tumor microenvironment contributes significantly to cancer progression. A relatively high proportion of malignant tumor tissues exhibit increased glycolytic properties, including HCC [30]. Che et al. reported that cholesterol synthesis accelerated cholesterol ester production and reduced triglyceride levels, thus accelerating hepato-carcinogenesis [31]. Highly expressed metabolic genes in HCC may accelerate metabolic dysfunction and tumorigenicity [32]. Therefore, elucidating the relevant metabolic pathways in HCC is crucial for prevention and treatment. Metabolic reprogramming studies have been conducted on glycolysis and the tricarboxylic acid cycle [33]. Many metabolic pathways occur in tumor cells, including fatty acid, glutamine, serine and cholesterol metabolism [34]. Numerous key enzymes in glycolysis are significantly upregulated in ovarian, pancreatic, breast and prostate cancer, as well as in osteosarcoma and melanoma [35]. Understanding the metabolic pathways that are disrupted in cancer can help researchers predict potentially responsible cells in tumor samples and gain insight into the disease etiology. For instance, Bénéteau et al. illustrated that inhibiting glycolysis could transform conventional tolerogenic cancer cells into immunogenic ones, thus providing a novel approach for immunogenic chemotherapy [36]. In the present study, we sought to establish a metabolic classification of HCC. Four specific subgroups were identified based on glycolytic and cholesterogenic pathways that significantly influenced survival.

Glycolysis promotes tumor progression, immune escape and drug resistance. Glycolysis can reduce dependence on oxygen and replenish tumor cells quickly. The intermediate products of glycolysis can be transferred to the pentose phosphate pathway and other pathways for protein, nucleic acid and lipid synthesis to meet the anabolic and metabolic requirements for rapid tumor cell proliferation [37]. Active glycolysis can reduce the permeability of the outer membrane of the mitochondria, allowing tumor cells to resist cell death [38]. Glycolysis produces large amounts of lactic acid, resulting in an acidic environment that is conducive to tumor invasion and immune escape [39]. Tumor glycolysis is associated with changes in intracellular signaling pathways induced by a variety of oncogenic and/or tumor-suppressor genes. For example, the long non-coding RNA Ftx was found to promote aerobic glycolysis and tumor aggression by inducing the PPARγ pathway in HCC [40]. interestingly, Interestingly, in our study, the HCC subtype with higher glycolytic gene expression but lower cholesterogenic gene expression seemed to be both aggressive and sensitive to chemotherapy, as we observed survival benefits in this subgroup. Our results suggest that there are multiple metabolic phenotypes associated with glycolysis and cholesterol synthesis in HCC.

The liver is an important site of lipid metabolism, and cholesterol levels rise significantly in liver cancer cells. The activation of extracellular signal-regulated kinase in liver cells can inhibit the expression of the rate-limiting enzyme in cholesterol metabolism, thus inhibiting bile acid synthesis and inducing cholesterol accumulation [41]. Cholesterol metabolites or intermediates have been reported to promote the growth of cancer cells [42]. We confirmed the involvement of cholesterogenesis in the progression and classification of HCC by demonstrating that cholesterol synthesis genes were highly expressed in the tumor tissues of some patients and were associated with a poorer prognosis. Previous reports have indicated that statinstatins suppress proliferation and induce apoptosis in HCC cells and improve the prognosis of HCC patients [43, 44]., further illustrating the influence of metabolic reprogramming on HCC tumorigenesis.

We found that cholesterogenic gene expression correlated negatively with *TP53* expression and positively with *MYC* expression. Thus, abnormal expression of *TP53* and *MYC* may promote the malignant process of tumors by enhancing cholesterol synthesis and altering cholesterol use. In addition, by detecting mutated genes in metabolic pathways, we found that the MPC complexes regulating pyruvate flux were mutated and abnormally expressed in HCC, suggesting that changes in MPC are associated with HCC progression.

It is important to note that the correlation between glycolysis and cholesterol synthesis has been validated in various cancers. Our in-depth investigation demonstrated that the mutation of different metabolic genes and the expression of specific enzymes contribute to the unique metabolic characteristics and clinical prognoses of different cancer types. HCC metabolic typing based on metabolic reprogramming may provide important information to enable clinicians to select treatments, predict the potential response, anticipate treatment resistance and foresee the likely outcomes. Activating or inhibiting particular metabolic pathways may be a useful therapeutic strategy to prevent HCC progression.

## MATERIALS AND METHODS

### HCC dataset acquisition and processing

The HCC datasets and all corresponding clinical data were downloaded from the data portal of TCGA (Illumina HiSeq Systems; https://cancergenome.nih.gov/) and the ICGC (https://www.icgc.org) [45]. We also downloaded standardized RNA sequence data for all 423 available cases from TCGA and for 237 samples from the ICGC data portal. We used human genome reference sequence GRCh37 from the Genome Reference Consortium [46]. Samples from the ICGC were filtered to exclude those labeled as cell lines, xenografts, metastatic, normal or non-laser microscopy enrichment. After excluding these samples, we downloaded somatic mutational data (CNVs, SNVs and indexes) for all the screened samples.

### Analysis of RNA sequence data

We normalized the RNA levels in each sample using the Transcripts Per Million algorithm and log-transformed log_10_ ((normalized count*1e^6145^) +1). To identify significantly differentially expressed RNAs, we used a standard screen of a log_2_ fold change ≥ 1. All samples were screened to exclude those with tumor contents < 30% [47].

### Batch profile estimation and correction

**Figure 6:**
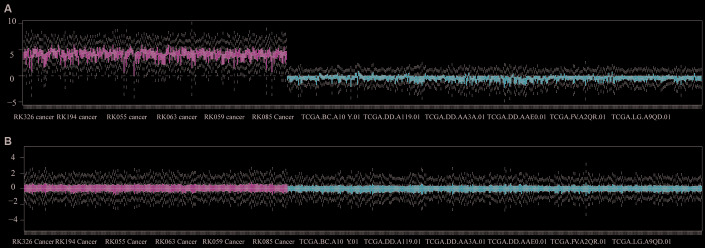
**Batch correction of queued datasets from TCGA and ICGC.** (**A**) Gene datasets were validated before normalization. (**B**) Gene datasets were illustrated after normalization.

We analyzed data from TCGA and ICGC cohorts, applied a normalization strategy and removed unwanted variation as a correction. Batch corrections were performed in each cohort using a gene localization scale (Figure 6).

### Classification of metabolic gene subgroups

The Molecular Signatures Database is critical for insightful interpretation of large-scale genomic data [48]. We used the "REACTOME GLYCOLYSIS" (n = 29) to identify the glycolytic subgroup and the "REACTOME CHOLESTEROL BIOSYNTHESIS" (n = 72) to identify the cholesterogenic subgroup. Subtypes were separated based on the consistent clustering of glycolytic and cholesterol-generating genes in ConsensusClusterPlus v1.38, and their statistical significance was assessed with SigClust (parameters: 160 reps=100, p Item=0.8, p Feature=1) [49]. Ward.D2 and the Euclidean distance were respectively used as clustering algorithms and distance measurements (k = 5 for glycolytic and cholesterogenic genes in resected and metastatic LIHC samples [n = 610), the cluster1]), and clusters 1-5 were validated. All patients were divided into four groups based on the median levels of glycolytic and cholesterol-generating genes in each sample: the quiescent group (GLYCOLYSIS ≤ 0, CHOLESTEROL ≤ 0), the glycolytic group (GLYCOLYSIS > 0, CHOLESTEROL ≤ 0), the cholesterogenic group (GLYCOLYSIS ≤ 0, CHOLESTEROL > 0) and the mixed group (GLYCOLYSIS > 0, CHOLESTEROL > 0). For each gene cluster, the ratio of glycolytic genes and cholesterol-generating genes was calculated. Gene clusters containing > 90% CHOLESTEROL genes or > 30% GLYCOLYSIS genes were considered as “core” gene clusters.

### Classification of pre-existing HCC subgroups

Consistent clustering was applied to classify samples based on the common tumor subtypes studied by Hoshida et al. [50], Budhu et al. [51] and Chew et al. [52]. The Hoshida subgrouping was based on the 186 genetic characteristics in the original publication, the Budhu subtyping was based on the 17 genetic characteristics in the original paper, and the Chew subtyping was based on the 14 genes in the original study. During each subtype classification, the samples were consistently clustered based on the genes of each classifier, and then semi-automatic subtype assignment was applied.

### RNA expression analysis of *MPC1/2*

We used RNA-seq data to identify gene sets that correlated positively or negatively with *MPC1/2* (Spearman correlation analysis), and performed BH correction for multiple test corrections. A significant correlation was established between two gene sets based on an adjusted p value < 0.01. A correlation coefficient of r > 0 was used to establish a positive correlation of a gene with *MPC1/2*, while a correlation coefficient of r < 0 was used to establish a negative correlation. To determine the pathway enrichment of genes that correlated positively and negatively with *MPC1/2*, we performed a comprehensive gene set enrichment analysis on the two sets of genes.

### Calculation of tumor content

The purity of each tumor sample was limited due to the presence of various immune cells surrounding the tumor cells, as well as other cells in the tumor microenvironment. We estimated the tumor purity of the HCC samples using an R package.

### Mutation analysis of HCC genes

We identified and analyzed gene sequences from the human genome assembly GRCh37/hg19 [53]. In order to identify oncogenic molecular events in the HCC metabolic subtypes, we investigated the frequency of SNVs, INDELs and CNVs in commonly mutated HCC genes [54] and explored their relationship with the HCC metabolic subtypes. With respect to tumor ploidy, DNA fragments with copy statuses ≥ 3 and ≤ 1 were considered amplified and deleted, respectively. Based on a previous study [55], we screened HCC copy number events with at least 10 support probes and fragment averages > 0.2 (amplified) or < -0.2 (deleted). The coordinates of the copy number events were mapped to the gene coding region with Bedtools v2.26, and the SNVs and CNVs of each gene were tested with a contingency analysis. The selected genes in each subgroup were tested, and Fisher’s exact test was used to determine whether there was a loss-of-function mutation or copy number amplification/deletion in each subgroup. BH correction was applied to the resulting p values.

### Pan-cancer RNA-seq analysis

We downloaded the Transcripts Per Million data of all pan-cancer samples and screened the samples according to cancer type. There were at least 100 samples after the preliminary screening, and there were 26 matched cancer types. Logarithmic conversion, batch correction, grouping and cluster analysis of RNA expression values were performed as described above. For each gene cluster, we calculated the ratio of glycolytic and cholesterol-generating genes. Gene clusters containing > 90% cholesterogenic genes or > 30% glycolytic genes were considered as “core” gene clusters. For cancer types with multiple core clusters in the same gene set, the most homogeneous cluster was considered to be the core cluster. Cancer types that did not have at least 75% homogeneous core glycolytic and cholesterogenic clusters were omitted from further analyses (specifically: bladder urothelial carcinoma, breast invasive carcinoma, colon adenocarcinoma, head and neck squamous cell carcinoma, kidney renal papillary cell carcinoma, acute myeloid leukemia, rectum adenocarcinoma, skin cutaneous melanoma, testicular germ cell tumors, thyroid carcinoma, thymoma and uterine corpus endometrial carcinoma (Supplementary Figure 1)). The metabolic subtypes of patients with each cancer type were determined based on the median values of the respective core glycolytic and cholesterol-generating genes.

### Survival analysis of HCC patients

Kaplan-Meier diagrams were generated using the R packages "survival" v.2.4.2 and "survminer" v.0.4.2 [56]. Patients with a total survival of less than one month were removed from the survival analysis.

### Statistical analysis

We used SPSS 23.0 software (IBM Corp., Armonk, NY, USA) and GraphPad Prism 7 (San Diego, CA, USA) to analyze the data [57]. Pearson correlation coefficients were used to express the linear correlations between pairs of variables. Kaplan-Meier and log-rank tests were used to analyze patient survival. R Studio was used to obtain the best cut-off value for each gene and survival curve. Two-sided p values < 0.05 were considered significant.

## Supplementary Material

Supplementary Figure 1

Supplementary Table 1

Supplementary Table 2
