# Supplementary material for: Alterations in glycolytic/cholesterogenic gene expression in hepatocellular carcinoma
Source: Aging (Albany NY). 2020 Jun 1;12(11):10300–16. doi: 10.18632/aging.103254 (PMC7346031; doi:10.18632/aging.103254)
Supplement: Supplementary Figure 1 [file aging-12-103254-s002..pdf]

SUPPLEMENTARY FIGURE

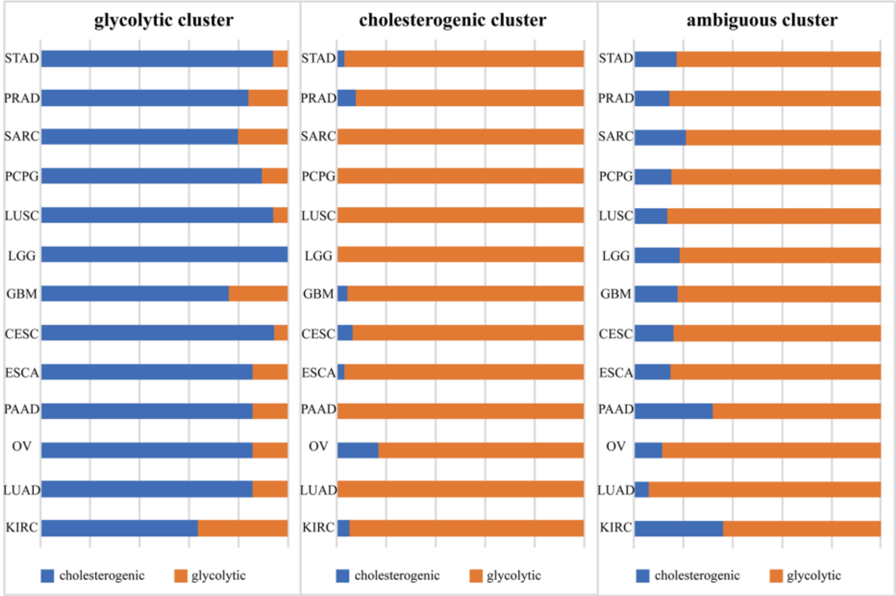

Supplementary Figure 1. Glycolysis- and cholesterol-related core gene clusters in 13 cancer types.
