# Supplementary material for: Alterations in glycolytic/cholesterogenic gene expression in hepatocellular carcinoma
Source: Aging (Albany NY). 2020 Jun 1;12(11):10300–16. doi: 10.18632/aging.103254 (PMC7346031; doi:10.18632/aging.103254)
Supplement: Supplementary Table 1 [file aging-12-103254-s003..pdf]

SUPPLEMENTARY TABLES

Supplementary Table 1. Extent of various LIHC subtypessubtypes and metabolic phenotypes in each sample.

Row Labels	Count of Hoshiba_groups	Count of Budhu_groups	Count of Chew_groups
Cholesterogenic	132	132	132
Glycolytic	146	146	146
Mixed	167	167	167
Quiescent	163	163	163
Grand Total	608	608	608

Count of Chew_groups	Column Labels			Count of Chew_groups	Column Labels		
Row Labels	Good	Poor	Grand Total	Row Labels	Good	Poor	*p* value
Cholesterogenic	37	95	132	Cholesterogenic	28.03030303	71.969697	0.22701108
Glycolytic	81	65	146	Glycolytic	55.47945205	44.520548	0.0156542
Mixed	73	94	167	Mixed	43.71257485	56.2874252	0.38750472
Quiescent	61	102	163	Quiescent	37.42331288	62.5766871	
Grand Total	252	356	608				

Count of Budhu_groups	Column Labels			Count of Budhu_groups	Column Labels		
Row Labels	Mam	Mim	Grand Total	Row Labels	Mam	Mim	*p* value
Cholesterogenic	34	98	132	Cholesterogenic	25.75757576	74.2424242	1
Glycolytic	52	94	146	Glycolytic	35.61643836	64.3835616	0.12422132
Mixed	29	138	167	Mixed	17.36526946	82.6347305	0.22402215
Quiescent	40	123	163	Quiescent	24.5398773	75.4601227	
Grand Total	155	453	608				

Count of Hoshiba_groups	Column Labels			Count of Hoshiba_groups	Column Labels		
Row Labels	Good	Poor	Grand Total	Row Labels	Good	Poor	*p* value
Cholesterogenic	46	86	132	Cholesterogenic	34.84848485	65.1515152	0.02223115
Glycolytic	65	81	146	Glycolytic	44.52054795	55.4794521	0.39599375
Mixed	50	117	167	Mixed	29.94011976	70.0598802	0.00243795
Quiescent	85	78	163	Quiescent	52.14723926	47.8527607	
Grand Total	246	362	608				

Please browse Full Text version to see the data of Supplementary Table 2.

Supplementary Table 2. Consistent cluster analysis of glycolytic and cholesterogenic gene expression in 26 cancer types (tumor content $\geq$ 30%).
